# Experiences of stigma and access to care among long COVID patients: a qualitative study in a multi-ethnic population in the Netherlands

**DOI:** 10.1136/bmjopen-2024-094487

**Published:** 2025-06-06

**Authors:** Gertrude Nsorma Nyaaba, Marieke Torensma, Maria Ingeborg Goldschmidt, Marie Nørredam, Ellen Moseholm, Brent Appelman, Mikael Rostila, Peter Tieleman, Sara Biere-Rafi, Maria Prins, Erik Beune, Charles Agyemang

**Affiliations:** 1 Faculty of Medicine, Health and Social Care, Canterbury Christ Church University, Canterbury, UK; 2 Public and Occupational Health, Amsterdam UMC Location AMC, Amsterdam, The Netherlands; 3 Infectious Diseases, Copenhagen University Hospital Hvidovre, Hvidovre, Denmark; 4 Danish Research Centre for Migration, Ethnicity and Health, Section for Health Services Research, Department of Public Health, Faculty of Health and Medical Sciences, University of Copenhagen Faculty of Health and Medical Sciences, Copenhagen, Denmark; 5 Clinical Medicine, Faculty of Health and Medical Sciences, University of Copenhagen Faculty of Health and Medical Sciences, Kobenhavn, Denmark; 6 Centre for Infection and Molecular Medicine, Amsterdam University Medical Centers, Amsterdam Institute for Infection and Immunity (AII), Amsterdam UMC Locatie AMC, Amsterdam, The Netherlands; 7 Centre for Health Equity Studies, Stockholm University/Karolinska Institutet, Stockholm University, Stockholm, Sweden; 8 Department of Public Health Sciences, Stockholm University, Stockholm, Sweden; 9 C-support, Amsterdam, The Netherlands; 10 Infectious Diseases, Amsterdam UMC Locatie AMC, Amsterdam, The Netherlands; 11 Infectious Diseases, Public Health Service of Amsterdam (GGD), Amsterdam, The Netherlands; 12 Division of Endocrinology, Diabetes, and Metabolism, Department of Medicine, The Johns Hopkins University School of Medicine, Baltimore, Maryland, USA

**Keywords:** QUALITATIVE RESEARCH, Long COVID, Public health, Delivery of Health Care, Integrated, Health Services, Health Equity

## Abstract

**Abstract:**

**Objective:**

This study explored the experience of stigma and access to healthcare by persons with long COVID from the majority Dutch and two ethnic minority populations (Turkish and Moroccan) living in the Netherlands.

**Design:**

This was a cross-sectional qualitative study that employed inductive and deductive thematic approaches to data analysis using MAXQDA.

**Setting and participants:**

Between October 2022 and January 2023, 23 semi-structured interviews were conducted with participants of Dutch, Moroccan and Turkish ethnic origins with long COVID living in the Netherlands. Participants were men and women aged 30 years and above.

**Results:**

Guided by the concepts of stigma and candidacy, the findings are structured according to the broader themes of stigma and access to care. The findings show that people with long COVID suffer self and public stigma resulting from the debilitating illness and symptoms. Especially among Turkish and Moroccan ethnic minority participants, strong filial obligations and gendered expectations of responsibility and support within their communities further worsen self-stigma. This experience of stigma persisted within healthcare where lack of information and appropriate care pathways led to feelings of frustration and abandonment, especially for participants with pre-existing health conditions which further complicate candidacy. Under the access to healthcare theme, the findings show multiple challenges in accessing healthcare for long COVID due to several multifaceted factors related to the various stages of candidacy which impacted access to care. Particularly for Turkish and Moroccan ethnic minority participants, additional challenges resulting from limited access to information, pre-existing structural challenges and experience of stereotyping based on ethnicity or assumed migrant identity by health professionals further complicate access to health information and long COVID care.

**Conclusions:**

The findings call for urgent attention and research to identify and coordinate healthcare for long COVID. There is also a need for accessible, informative and tailored support systems to facilitate patients’ access to information and care pathways for long COVID. Providing tailored information and support, addressing the various barriers that hinder optimal operating conditions in healthcare and leveraging on social networks is crucial for addressing stigma and facilitating candidacy for persons with long COVID towards improving access to care.

**Strengths and limitations of the study**

- A key strength of the study is that it included vulnerable and hard to reach populations that are often overlooked in healthcare and health research.
- The study population might not fully capture the range of perspectives of ethnic minority perspectives (eg, sub-Saharan African, South and Northern Asian) on long COVID and the larger study population with long COVID as debilitating illness and language barriers hindered some eligible persons from participating in the study. As such, study findings may not apply to other ethnic Dutch, Turkish or Moroccan persons with experiences of debilitating long COVID symptoms living in the Netherlands.
- Although most study participants were recruited from the C-support foundation, which requires some language and digital literacy skills that can be challenging for older populations or persons from low socio-economic status, a key strength of this study was the use of multiple modes of recruitment to identify and recruit additional study participants from previous COVID-19 community education sessions via two Mosques. This sampling strategy ensured variation in ethnic origin, gender, educational level, migration status and Dutch language proficiency.
- Some participants may also have experienced recall bias or might have omitted details, resulting from the symptoms associated with long COVID. To facilitate all participants’ recall efforts during the interview,^51^ participants were asked, before the interview, to develop a timeline, marking the onset of their symptoms, interactions with the healthcare system and meaningful experiences. These were used together with the topic guide for the interviews.
- Lastly, the use of member checking of summaries of transcripts via phone call ensured that participants who could not speak Dutch were included.

## Introduction

 The COVID-19 pandemic had multiple impacts on people’s health, particularly for persons experiencing long-term post COVID-19 complications.^1 2^ Literature on COVID-19 and its impact is growing,^3^,^6^ but qualitative literature on post COVID complications such as long COVID is limited. Long COVID can affect anyone and at any age regardless of health status.^7^ Evidence also shows that ethnic minority populations have higher rates of SARS-CoV-2 infection and subsequent hospitalisations in Europe and the USA with a higher risk of long-term health consequences following COVID-19 hospitalisations compared with the majority population.^8^,^14^ Recent studies conducted in Canada and the USA further show that non-hospitalised persons who tested positive for SARS-CoV-2 infection were significantly less likely to self-report long COVID symptoms compared with previously hospitalised persons.^15 16^ Non-hospitalised populations, especially from ethnic minority populations, generally have less access to care and rehabilitation programmes with limited literature on the role of ethnicity as a risk factor for long COVID and how it shapes access to care. Addressing the healthcare needs of people with long COVID therefore calls for a comprehensive understanding of the lived experiences of people with long COVID, especially among ethnic minority populations, to inform tailored interventions to address their healthcare needs.

Long COVID is a term commonly used to describe symptoms of COVID-19 that persist beyond the acute illness.^17^ The WHO reports that it occurs in individuals with a history of probable or confirmed SARS CoV-2 infection, who continue to experience symptoms 3 months after the onset of COVID-19 with symptoms lasting a minimal duration of at least 2 months, which cannot be explained by an alternative diagnosis.^18^ Although symptoms greatly differ between individuals, with over 200 reported symptoms with wide prevalence; common symptoms include weakness, general malaise, post-exertional malaise, fatigue and concentration impairment.^19^,^21^ Symptoms may be new onset following the initial recovery from the acute phase of COVID-19 or persist from the initial illness, fluctuate or relapse over time and generally impact a person’s everyday functioning and reduce their quality of life.^18 20^

Previous studies show that 13.7% of non-hospitalised people continued to report complaints 3 months after testing positive for SARS-CoV-2, and 33–87% of patients who had been hospitalised reported persistent complaints in the UK.^22^,^25^ For ethnic minority populations living in high-income countries, a study shows that COVID-19 hospitalised patients with ethnic minority backgrounds are more likely than those of Dutch origin to report having long COVID; with significant differences in occurrence, nature of symptoms and duration of long COVID by migration background.^8^ Another study reported an increased risk of long COVID among ethnic minority populations compared with native Danes in a nationwide register-based cohort study with both hospitalised and non-hospitalised individuals.^26^ They called for further research to understand long COVID drivers and address care strategies among ethnic minority populations.

Several attributes of long COVID, such as its extended duration, the lack of a valid and uniform diagnostic test and effective treatment, render it a stigma-prone condition.^27^,^30^ Research on lived experiences in Italy shows that long COVID is stigmatised,^31^ with a UK study reporting that 75% of participants with suspected or confirmed long COVID reported often experiencing stigma.^32^ This experience of stigma can lead to poor health-seeking behaviour, which may exacerbate illness particularly for minority ethnic groups, due to aspects related to their identity such as race or ethnicity which can lead to the experience of ‘*double stigma*’.^2731^,^33^ *Stigma* is a discrediting quality, which diminishes the status of an individual^34^ and occurs in social interactions. It is, therefore, entirely socially constructed with multiple manifestations such as public stigma, self-stigma and structural/institutional stigma (*Section A,* Table 1).^34^,^36^ The fear of stigma is worsened by implicit biases within healthcare and social systems, which place minority ethnic groups at a disadvantage in accessing and receiving quality care.^37^

**Table 1:** Concepts of stigma and candidacy

**(A) Concept of stigma**	
Concept/stage	Definition
Self-stigma	the social and psychological impacts of having a stigma and encompasses both the experience and anticipation of encountering stigmatising behaviour (felt stigma) and the diminished self-worth and feelings of shame that often accompany a stigmatising condition (internalised stigma)
Public stigma	the social and psychological reactions towards individuals with a stigmatised condition or attribute.
Structural/institutional stigma	Legitimisation and perpetuation of a stigmatised status by society’s institutions and ideological systems.^38^

(B) Candidacy concept	
Stage	Description
(1) Identification of candidacy	How individuals decide their symptoms require medical attention.
(2) Navigation	Individuals knowledge of and ability to gain entry to appropriate services.
(3) Permeability of services	Ease of entry into the health system determined by system's internal factors, such as cultural alignment or the availability of care pathways for specific conditions.
(4) Appearances at health services	The process of asserting candidacy during interactions with health professionals.
(5) Adjudications	Professional judgements about an individual’s candidacy, influencing subsequent treatment.
(6) Offers and resistance	How follow-up services may be accepted or rejected by the patient depending on their appropriateness.
(7) Operating conditions and the local production of candidacy	Locally specific factors that influence interactions between patientd and healthcare professionals and develop over time.

Sources: References 33,40.

Moreover, access to care influences an individual’s ability to effectively use health services or informal care, which encompasses both supply and demand factors.^38^ It is influenced by the concept of *candidacy,* which is a dynamic seven-staged process (Section B. table 1) by which people determine their eligibility for health services, through continuous negotiations with healthcare professionals.^39^,^41^ The candidacy model is particularly useful for exploring and understanding how vulnerabilities arise in relation to healthcare access and how these are influenced at multiple levels and differ between groups and across multiple intersections.^40 41^ The situation may be compounded for persons less familiar with health services, especially among ethnic minority populations where health literacy is generally lower.^39^

This study, therefore, draws on the concepts of *stigma* and *candidacy*^33 38^ to explore people’s lived experiences of long COVID, focusing on the experiences of stigma and access to care of ethnic minority populations in comparison with the majority (Dutch) population in the Netherlands.

## Methods

A qualitative approach was used to conduct semi-structured interviews using questions and prompts originating from the theoretical framework on stigma and candidacy^41 42^and patients self drawn timelines to guide participants in narrating their illness and patient journeys. As a phenomenological study, this enabled participants to share their illness experiences and touch on sensitive illness experiences while allowing the researcher to explore known and unexpected outcomes.^43^,^46^

### Study population

Persons with confirmed initial SARS CoV-2 infection by means of PCR or antigen testing (self-testing), who continued to experience more than one symptom 3 months after the onset of COVID-19, lasting for at least 2 months^47^ and for whom such symptoms had an impact on their daily lives were included. Persons with differential diagnosis (not SARS CoV-2 infection) or symptoms were purposively excluded. Persons with Dutch, Moroccan and Turkish ethnic origins were purposively included, as they were among the most affected groups by SARS CoV-2 infection in the Netherlands.^8 11 12^ Identification of ethnic origin was done based on the classification system of Statistics Netherlands, ie, persons are considered of Dutch origin when they and both their parents are born in the Netherlands, while persons are considered to have a Turkish or Moroccan origin if they, or at least one of their parents, is born in Türkiye or Morocco.^48^ In qualitative research, sample sizes are guided by the principle of saturation.^43 44 49^ Hennink and Kaiser (2022) established that saturation of in-depth interviews was reached at 9–17 interviews.^50^ Since there is a considerable amount of heterogeneity in this study sample and the informational richness of the interviews depended on the cognitive functioning of participants, we anticipated that a sample size higher than the established number of interviews was needed to reach saturation. Consequently, and to ensure variation in ethnic origin, gender, educational level, migration status and Dutch language proficiency towards reaching data saturation, a purposive sampling approach was used to include a minimum of twenty (20) participants.

### Setting and r*ecruitment*

Study participants were recruited using two approaches. First, they were recruited from among persons with long COVID registered with the C-support foundation in the Netherlands. C-support is a state-financed organisation that provides free information, guidance and advice to patients with long COVID, across the Netherlands.^51^ Persons with long COVID registered with C-support were approached by C-support via email containing study information and asking their interest in participating in a scientific study. For interested persons, C-support requested permission to share their contact details with the research team. The shared first author (MT) contacted potential participants (n=20) by phone to check eligibility criteria (table 2), introduce the research and research procedures and answer questions from potential participants. Once eligibility and willingness to participate were established, a date, time and place were scheduled for semi-structured interviews to be conducted (n=20). Second, two research assistants (RAs) with Turkish, Moroccan, Berber and Arabic language proficiency who had access to Turkish and Moroccan communities approached 9 persons with long COVID with limited Dutch language proficiency who were not registered with C-support. These persons had previously attended community education sessions on COVID-19 at two Mosques. Seven of the 9 persons were then included after establishing eligibility and willingness to participate. Out of the total of 27 persons eligible and willing to participate, four persons withdrew due to increased burden of their long COVID symptoms (n=2), family circumstances (n=1) and concerns regarding audio recording of the interview (n=1), resulting in a study sample of 23.

**Table 2:** Inclusion criteria

Question	Response	Action
When was your initial COVID infection?	Date	
Do you continue to have any symptoms?	Yes/No	If no—excludeIf yes—ask next question
Do your symptoms bring about a significant change in your daily functioning with regard to housekeeping, care, studies or work and/or affect interpersonal relationships?	Yes/No	If no—excludeIf yes—include

To facilitate participants’ recall efforts during the interview,^52^ they were asked to develop a timeline, marking the onset of their symptoms, interactions with the healthcare system and meaningful experiences, among others prior to the interview. These individual timelines were used together with the topic guide to facilitate the interviews. All participants were sent a text message via mobile phone the day before the interview to confirm the appointment and ensure their health allowed for the interview to take place. Post-interview, in line with good research practice, all participants again received phone text messages to check up on any health impacts experienced following their interviews to provide some guidance and signpost to appropriate healthcare for further support. Participants, however, reported no concerns. Subsequently, all participants received a reimbursement (€15, - gift card) for their participation.

### Data collection

Interviews were conducted between October 2022 and January 2023. Out of the total of 23 individual interviews conducted, 20 interviews were conducted face to face (in private at participants’ homes), while three interviews were conducted by MT online using video conferencing software. MT conducted 17 interviews in Dutch, while each of the two RAs conducted three interviews in Berber, Moroccan Arabic or Turkish. A semi-structured interview guide, which included questions on access to care and the experience of stigma originating from the theoretical framework on stigma and candidacy^35 38 40^ (online supplemental file 1), together with the individual timelines were used for all interviews. RAs translated the interview guide into Berber, Moroccan Arabic, and Turkish.

Interviews lasted between 45 to 90 min, were audio recorded with participants’ consent and transcribed verbatim into Dutch. Interviews conducted in Turkish, Berber, Moroccan or Arabic were transcribed and translated into Dutch by the RAs. A 1–2-page summary of the transcript was shared with each participant, accompanied by a follow-up phone call to discuss the summary with participants. Given the considerable amount of heterogeneity within the sample, 23 interviews enabled us to reach saturation with no repeat interviews conducted. Participant characteristics are presented in Table 3 below.

**Table 3:** Participant characteristics

Characteristics	Number
(Ethnic) Background (first*)*	
Turkish (Tu)	9 (7 (first generation))
Moroccan (Mo)	7 (5 (first generation))
Dutch (Du)	7
Gender	
F	18
M	5
Age category	
30–39	4
40–49	5
50–59	10
60–69	3
70–79	1
Educational level	
Higher educational level (HBO/WO:)	9
Mid educational level (MBO2 t/m MBO4)	8
Low educational level (MBO1, VMBO, onderbouw HAVO/VWO (CBS categories))	6

### Data analysis

Transcripts were thematically coded using MAXQDA 12 (maxqda.com/help-max20/welcome), a qualitative data analysis software. Data analysis was done in phases composed of familiarisation with the data; generating initial codes; searching for themes; reviewing themes to explore commonalities and differences; defining and naming themes; and producing the manuscript.^53^

MT deductively developed the initial coding framework based on the research question and theoretical framework. MT and Nazli Lale-Kahraman (a junior researcher) both coded, compared and discussed 8 transcripts using the initial coding framework, which was revised to include inductive codes emerging from the transcripts. The final coding framework was then used by MT and Nikita Hensen (a junior researcher) to code all remaining transcripts. MT inductively generated initial themes from the coded data, discussed and revised them with EB to explore commonalities and differences between ethnic minority groups and the majority (Dutch) population. Relevant participant quotes were then translated by (www.DeepL.com/Translator (free version)) from Dutch to English for the manuscript development.

### Patient and public involvement in research

Patients and the public were not explicitly involved in the design of the study but contributed to the research study as participants giving voice to persons/people with long COVID, to share their experiences on stigma and accessing healthcare for long COVID to inform health pratice and policy. In addition, C-support, which is a Dutch Ministry of Health, Welfare and Sports-commissioned foundation, functioning as an informal national registry of persons with long COVID in the Netherlands, providing support and advice to persons with long COVID and sharing emerging knowledge on long COVID with healthcare professionals will contribute to the dissemination of the study findings with and to long COVID patients.

### Ethics approval and consent to participate

The medical ethical committee of the Amsterdam University Medical Centres/University of Amsterdam declared this study declared this study as nWMO, according to the Dutch Medical Research Involving Human Subjects Act W22_198 # 22.246 (https://wetten.overheid.nl/BWBR0009408/2022-07-01).

Conducted in accordance with the COnsolidated criteria for Reporting Qualitative research (COREQ) Checklist (online supplemental file 3), this study also obtained written and oral (recorded) informed consent from all participants.

## Results

The results are presented under the two main themes arising from the conceptual framework with sub-themes and emerging codes. Table 3 shows that the majority of study participants were female (n=16/23), several participants were aged between 50–59 years (n=10) and with higher education (HBO/WO) (n=9). For ethnic minority participants, the majority were first-generation migrants (n=12 out of 16). Selected quotes are embedded in the results, and additional participant supporting quotes labelled Tu (Turkish), Mo (Moroccan) and Du (Dutch) are also presented as online supplemental file 2.

### Stigma

Participants from both the majority (Dutch) population and Turkish and Moroccan ethnic minority populations reported that the initial understanding and empathy for their illness atrophied over time as they relapsed or continued to experience long COVID symptoms.

#### Experience of public and self-stigma

Participants experienced public stigma mostly in the questioning of their long COVID symptoms during social interactions with other people, often feeling and being referred to as ‘*aansteller’* (Dutch for ‘*poser’*) and receiving unsolicited advice on the causes and solutions for their symptoms.

I notice that in the beginning everyone was understanding, but as it takes longer the understanding diminishes. And then… maybe they think you are doing it on purpose, or there’s something else going on… Among colleagues, or friends, then one says maybe you have burnout, maybe you are depressed, then you get that kind of thing. I feel like everyone just says things. Participant 6—TU

Regarding self-stigma, participants reported experiencing a loss of identity and purpose that contributed to lower self-worth and feelings of depression (internalised sigma). Only a few participants reported finding meaning in their illness through their contribution to research or perceiving their illness as a test of their faith, which protected them from feelings of low self-worth. While most participants reported this internalised stigma, they did not report any anticipation of encountering stigmatising behaviour (felt stigma).

Participants reported variations in the severity and variety of debilitating long COVID symptoms they experienced, which caused them to feel low self-worth. They explained that their symptoms caused prolonged absences from work and family life, causing them to experience feelings of guilt and shame and, at times, fear of being seen as using their ill-health as an excuse to not work or contribute to the family (internalised stigma). An ethnic minority participant shared that such feelings were worsened by strong filial obligations and gendered expectations of responsibility and support within their communities. The participant acknowledged that such expectations potentially occur in other cultures and across genders.

I think you have…, lost your dignity, your independence, but also that at some point you can no longer take care of your family. And I'm not talking about the financial stuff, but the attention to your kids, a little bit of homework, the worry, reading letters to them …Yes you have lost your pride. You are a man… if you can't take your responsibility, carry it, execute it, yes then that is not nice……. you are just talking about the Moroccan community, and the Turkish community, but I don't want to say that it is very different from the Dutch community. I think that every head of the house, whether it is a man or a woman, that it is the same for a woman who is single with the children…because are you positive, or were you really sick too…. I was ashamed to prove that, how can you be sick for so long?

I: And what helped in it to stop feeling that shame?

R: Nothing… I'm glad I was better…. Then it got better [after contact with company doctor]. Then I had that recognition. Participant 4—MO

#### Stigma within the context of healthcare

Among most participants, the lack of information on long COVID generally and from healthcare professionals and the lack of available care pathways and treatments for long COVID contributed to feelings of abandonment by the healthcare system. In addition, insecurities caused by long COVID symptoms, particularly for patients who (already) experienced an array of health problems, affected their healthcare encounters as they felt that their long COVID complaints were not taken seriously by healthcare professionals, making them feel frustrated.

The frustrating thing was that I was already struggling with word-finding problems. I already had a haze in my head, … with my last bit of energy, I drag myself over there [GP] to go and sort something out and then it felt like the door is then slammed in front of your face… Participant 7—DU

Some participants reported that the multiple and severe symptoms they experienced, without a clear cause, resulted in some healthcare professionals assuming a psychological origin/cause for their symptoms.

#### Role of informal care/support

Participants reported that contact with other persons with long COVID, for example, via (online) support groups made them feel recognised and reassured as persons in those groups recognised their symptoms and challenges, which helped alleviate their experience of stigma. They mentioned that they also received information on long COVID healthcare pathways and options available through support groups and friends.

I was really in a dip about myself, is this really corona or something else? But by reading the messages and the stories of those people, that’s where I had the most support, this whole long period. Because there were similarities of what you have and them. Then you think “oh, it’s real,” and several people have the same symptoms, like mine then. Participant 14—MO

#### Ethnic differences in experiences of stigma

While the experiences of stigma did not differ greatly between Turkish and Moroccan ethnic minority participants and Dutch participants, two ethnic minority participants reported difficulties accessing care which they related to their ethnicity. A participant explained that while proficient in the Dutch language, a speech impediment was one of his/her long COVID symptoms, which resulted in him/her sounding ‘*like a migrant*’ and hindered his/her ability to efficiently communicate in Dutch verbally. Another participant felt that the care she/he received from C-support made her/him feel heard, understood and cared for in comparison with the care received from the general practitioner (GP). When explicitly asked about the experience of double stigma, however, most Turkish and Moroccan ethnic minority participants rejected the notion of being stigmatised or discriminated against because of their ethnicity.

I'm not going to say it’s because of my background, [but] you'll never know. … the first time I got sick, and I had gone to the hospital, then I did feel it [stigmatised]… But after that, C-Support, for example, you're by phone, you did feel understood and that you were listened to…. I really don't dare say it… I think you're just either lucky or a little unlucky, that’s it. I don't want to think about them knowing that I'm mocro and that I'm not…. No. I do have that sometimes… because I'm wearing a headscarf outside… you can't do anything about it. Participant 14—MO

### Access to care

#### Identification of candidacy and navigation to care

Participants generally identified their complaints as requiring medical attention and consulted their GP early on (within two to 4 weeks) after their initial COVID-19 illness. For many participants, the identification of candidacy halted with the GP services, where they were advised to wait out the progression of their symptoms, often because little or nothing was known about appropriate care pathways for long COVID. As a result, some participants reported that when they were eventually referred for specialist services which were not covered by health insurance after the maximum number of consultations eligible for reimbursement, they incurred significant financial costs to access healthcare.

After repeated, and often frustratingly fruitless, visits to the GP, some participants reported refraining from seeking care. Some Turkish and Moroccan ethnic minority participants expressed frustration with the bureaucratic nature of the Dutch healthcare system, and in some instances, this was compounded by limited Dutch language proficiency, which further complicated this first step in navigating healthcare. Non-Dutch-speaking participants shared that they depended on their children to schedule their GP appointments and to serve as translators during their GP appointments. Several participants reported that they experienced social isolation, exacerbated by their debilitating long COVID symptoms, which further complicated their access to healthcare services. They reportedly succumbed to inadequate care, accepting symptoms and experiencing little control over their situation.

After 2 months I was still not the same, I had no strength in my legs, I couldn't concentrate for a very long time after that either and became forgetful… I don't have the strength anymore and no desire to do anything, I get tired easily… I don't know why the symptoms last so long…I go to the family doctor, but she can't give me anything, so I didn't go back… I'm fed up with it all, that’s why I don't want to go. Who will go with me? I have no one [patient becomes sad]. When my son got sick, he also felt alone and said we are so lonely, there is no one to take us to the doctor. … I wish I could speak something Dutch and not bother him. Then I would handle my own problems myself, but I don't speak Dutch at all. I always depend on other people, and I find that difficult, I'm ashamed of it, I don’t know if they want to do it or not. Participant 25—TU

Having to find information on their illness themselves exposed participants to potential misinformation and disinformation, mostly electronically and via social media. While most participants found access to the appropriate information for their illness difficult regardless of Dutch language proficiency, some Turkish and Moroccan ethnic minority participants further expressed concerns for other members of their communities who had little or no Dutch language proficiency. They explained that for such community members who are challenged by language barriers, access to information on long COVID is limited during encounters with their GPs, resulting in gaps in information received from GPs. They added that even when information is available digitally (such as by C-Support), access to that information is further obstructed for migrants such as older migrants who might not be literate or digitally literate.

The way they had it set up, you have to log in…. you have to be fairly self-reliant, if you want to know the system and log in and then make an appointment, you have to do all that yourself…they have one of those digital systems that you can only register with your DigiD. So, I think my mother-in-law can't do all that. … because she’s just not digitally savvy, if you're not self-reliant, or you're not digitally savvy… But suppose I hadn't known the way well either, then you do find yourself in a sort of 'loop' with each other, of how to find the right care. Participant 11—TU

I: There is a site on the Internet called C-Support, have you ever heard of it?

R: No I'm illiterate and don't know how to find this on my phone, I didn't study…I can ask my son or… Participant 19—MO

All participants expressed disappointment that GPs could not provide more direction to finding the cause or treatment for their illness. While participants were primarily responsible for navigation to appropriate care, there were great variations in the actions participants were able to undertake themselves, which related strongly to their ability to rely on and use social networks. Most participants, regardless of ethnicity, reported relying on their social networks to identify and access information and care for their illness and symptoms. Most participants requested organised support in coordinating and navigating to care to improve care for long COVID. Dutch-speaking participants also shared that C-support helped direct them towards care, informing them on specific requests for referrals, or available care within the vicinity of their homes. They mentioned various ways that enabled them to access C-support as they relied on social networks and digital literacy skills to be directed to C-support. Although there was no uniformity in access to C-support, Turkish and Moroccan ethnic minority participants who could not sufficiently communicate in Dutch or had limited digital skills reported not being aware of C-support.

And then someone in my network who had contact with his physio practice with a number of physio practices in other countries … And his friend is a good friend of mine, and she said “hey, go talk to my friend.” His is very similar to that… I did google, what could this [long COVID] be? … I don't know if my own GP had that knowledge at that time…. It was all so vague. So, I made an appointment with him [friend referral/health professional], sat down, explained my symptoms. And then he told me of well this is what we see, when people try to build up [recover], relapse … I think, “yes, this is it,” I recognize myself in this. Participant 7—DU

Only four participants (3 (DU), 8 (DU), 15 (TU), 18 (TU) reported having had access to a clear care pathway early on, that is, timely referral to long COVID rehabilitation for primarily physical therapy and occupational therapy from their GPs or company doctors. They explained that this was mainly dependent on their GPs’ awareness of current COVID-related issues and pro-activeness in accessing information to support them.

#### Permeability

In the Netherlands, GPs function as the main gateway to care, with most participants identifying several factors that complicated their ease of entry to accessing healthcare via the GP (see^54^ for a description of Dutch system).

For some participants, employment provided an opportunity to enter the healthcare system. They explained that after a few weeks of absence from work due to illness, a company doctor (*who looks specifically at the work environment, potential reintegration and is in contact with the GP only with the consent of the employee*) attended to them, which provided an opportunity to be heard and hear about long COVID and be diagnosed and referred for rehabilitative care. They noted that company doctors were more influential in their employment and could direct them to healthcare and advocate for longer recovery time from employers. While some participants added that the company doctor’s diagnosis of long COVID immensely relieved them, a few others reported that ill-informed company doctors hampered their access to care and recovery time.

I’m still very happy with my company doctor… I started seeing the occupational therapist, which has done me very well. And I was able to take myself seriously and I finally I thought that I can just be sick… the GP can't do very much for you… he doesn't have that much influence on you, on your work situation but the company doctor does…. my second company doctor did take me seriously, and confirmed that whole long COVID 100%, she said no doubt about it, you just have long COVID… I remember sitting there with her, I think I used up a whole box of tissues… she also knew a lot about long COVID, and that was also very nice. Participant 18—TU

While participants expected their GPs to be the connecting and coordinating link in their care appointments, because of the organisation of GP practices, where usually several GPs work in one practice, participants had to explain their complaints repeatedly in case of replacement GPs who they regarded as less informed and less responsive to addressing their long COVID symptoms. Participants felt this affected the quality of healthcare they received as they sometimes felt less confident to speak up or demand care because of a lack of familiarity with replacement GPs which, added to limited consultation time, did not allow for familiarity and a comprehensive discussion of their multiple and varied symptoms.

Unfortunately, my own family doctor was not there. So, another GP explained what was there. And he also started telling me things about long COVID, all of which I already knew. Because by then I had already started at [name rehab centre], June 20 (or July) …. I had this feeling that he had just learned something about long COVID… I just didn’t think it was good care…. Participant 17—MO

My family doctor was on vacation and there was a substitute family doctor. And he said I think you are just overworked, and here, you have the number of the psychologist, go talk to a psychologist. I said, I think it’s super sweet that you think of me so much, but I know myself, it’s not in that corner, this is different… I said well fine, but then I’ll wait until my own family doctor is back. Participant 7—DU

Most participants mentioned that double GP appointment slots did not provide sufficient time for consulting on their multiple symptoms, with some of them suggesting that their symptoms may not have been taken seriously, resulting in fewer referrals for specialist healthcare. Particularly for Turkish and Moroccan ethnic minority participants, although they expressed understanding of this set-up, this characteristic of the Dutch health system frustrated them, prompting them to contemplate seeking healthcare in their home countries or other less restrictive countries. An ethnic minority participant added that the restrictive nature of the Dutch health system meant longer waiting times despite the excessive cost of health insurance.

The Netherlands is a good country, all tightly regulated, we pay VAT for it too, all that insurance. But bureaucratic as hell… I sometimes go to a doctor in Morocco, because if I have something, yes, it’s not top notch, but when I go, they can check right away. And here you have to be referred three times then you have to wait 6 months if you're lucky. But you do pay every month, my wife and I, €400.00 a month for the insurance. And then you have to beg for a CT scan, … Because in Belgium you're faster in that respect too, in France too, in Spain too… the advice I would give, particularly to general practitioners, deviate from that 5-minute consultation hour. Take somebody seriously. Someone who has a complaint…If I have complaints, and you just give me time to only talk about one complaint, when I'm just really sitting with other complaints. Participant 4—MA

Another ethnic minority participant explained that because the demography of a neighbourhood determines the expertise that is available at the GP, at GP appointments, some health professionals presume that they cannot communicate in Dutch or they are not born in the Netherlands, which influences how they behave and act towards them. The participants therefore felt such care was not tailored to suit their health needs. Especially for participants who got ill early in the pandemic, knowledge of their GPs and other healthcare professionals on long COVID was key to facilitating access to care.

The treatment towards patients did change, yes…. migrant background, they may also be, say, 3rd or 4th generation. not everyone who comes in here was born abroad or speaks poor Dutch… You feel that in the treatment, how people talk to you, the behaviour of the people who work there. … things like that are signals of I need to change GPs…maybe I don’t fit into the target group of that general practice anymore. Participant 6—TU

Most participants reportedly organised care for long COVID themselves by exploring possibilities for care electronically (online) prior to visiting their GPs to ask for a referral. Participants referred for long COVID rehabilitation therapies such as physical therapy, occupational therapy and speech therapy experienced challenges with coordination of their healthcare. A few participants who had been referred to various healthcare specialists reported feeling discouraged by the lack of follow-up appointments from healthcare professionals or specialists. While Dutch-speaking participants reported being referred for different therapies, most participants with limited Dutch communication proficiency reported less contact with the healthcare system. This implies entry barriers, as most of the non-Dutch speaking participants had only seen their GP, with only a few of them being referred for the physical therapy trajectory.

#### Appearances and adjudications

When interacting with healthcare professionals, many participants shared having to advocate for themselves to get healthcare but often felt uncomfortable/unable to do so. Consequently, participants’ views regarding healthcare professionals’ judgement about their candidacy, influencing subsequent treatment for their illness, considerably differed (adjudications). Turkish and Moroccan ethnic minority participants with limited Dutch proficiency reported being less able to clearly describe their symptoms and access referrals to healthcare and depending on informal translations. They reported self-medicating to manage their symptoms while depending on family members with Dutch proficiency to advocate for them to access healthcare for their symptoms.

Similarly, Dutch-speaking participants also shared experiencing difficulties with asserting candidacy, especially when they were not equipped with information and sought direction from healthcare professionals on how to manage their symptoms. They reported that their symptoms limited their physical abilities and capacity to assert themselves during interactions with healthcare professionals.

I was not taken seriously. Because long COVID that was nonsense, the family doctor said that was nonsense, he referred me to the physio and the ergo, well they didn’t do anything either. He was very negative… I didn’t know it myself, my surroundings didn’t know it, and I thought myself am I posturing?… No, and I didn’t have the knowledge, I didn’t have the energy. Participant 18—TU

Some participants explained that they were not referred for any therapies by their GPs because of limited evidence-based solutions at the time and/or did not have adequate knowledge about long COVID symptoms and care pathways. Some participants who received referrals mentioned that these therapies did not often significantly address their symptoms. Adjudications did not differ by ethnicity or ability to communicate in Dutch.

For participants who faced such challenges and were aware of C-support, C-support equipped them with information on specific requests for referrals they could make.

And then I got to C-Support …I called them, I had an application there, and this lady who called and she knew exactly…. I said, what I was going through, and she had looked up a report, she said show this report to the GP. Participant 12—TU

#### Offers and resistance

Some participants reported incurring additional healthcare costs when they had exceeded the deductible allocated for their health insurance. Other participants explained that despite having health insurance, they had incurred additional costs for specific referral treatments for their long COVID symptoms, which had not been reimbursed. Other participants reported inadequate information about continuing treatment options covered by their health insurance, which resulted in them incurring additional costs due to gaps in treatment.

When I joined C-Support, I had already passed half a year. Because I was not reimbursed for physiotherapy. …, I went to the doctor, who then didn't think maybe physiotherapy would be useful, and nothing else was followed up…they said at C-Support, well it might be possible if you write a letter. Participant 11—TU

Some participants felt that the care they received did not appropriately address their symptoms and needs, as they did not feel strong enough to return to their normal duties when they were discharged and often experienced a flare-up of their long COVID symptoms. This resulted in them seeking healthcare all over again. Some participants expressed frustration with the limited healthcare professionals’ expertise regarding their illness and the inadequate coordination of care strategies to manage their symptoms. While some participants reported being misdiagnosed, others reported counterproductive effects of some of the therapies they received. These experiences were reported among all ethnic groups with key implications for access to and acceptability of follow-up services for participants.

## Discussion

This study draws on the concepts of *stigma* and *candidacy* (30, 32) to provide insights into the lived experiences of people with long COVID with a focus on stigma and access to care from the experiences of two ethnic minority populations (Turkish and Moroccan) and the majority (Dutch) population in the Netherlands. The study findings show that participants suffer self and public stigma resulting from the prolonged experience of debilitating symptoms and the lack of information and appropriate care pathways within the context of healthcare, which led to feelings of frustration and abandonment. In addition, all participants experienced general difficulties in accessing healthcare for long COVID resulting from several multifaceted factors related to candidacy. The lack of information, appropriate care pathways and care coordination from health professionals as key operating conditions for healthcare adversely impacted participants’ health encounters, resulting in participants' acceptance of long COVID symptoms and refraining from seeking or accepting follow-up care services. However, social networks were strong sources of information which enabled participants to build collective and individual candidacy. For some ethnic minority participants, candidacy was further complicated by limited Dutch language proficiency, digital and/or health literacy and experience of stereotyping based on ethnicity or assumed migrant identity by health professionals.

The findings show that the varied symptoms of long COVID and the challenges managing these symptoms made participants feel misunderstood and afraid of being perceived as pretending. Although they did initially attract public empathy, this atrophied over time, contributing to the experience of self and public stigma when symptoms persisted or reoccurred. These challenges lead to feelings of isolation and depression, which facilitated internalised stigma. This was more evident among ethnic minority participants where collective and gendered norms surrounding family responsibility led to feelings of shame, guilt and a loss of identity as illness challenges made it difficult to engage in social and/or economic obligations, leading to identity disruptions. Similar findings have been reported in other studies where the perceived inability of people affected by long COVID to effectively manage their symptoms or reduce the severity of their symptoms resulted in all forms of stigma and identity dilemmas.^27 31 32 55 56^

Within the candidacy framework, the process of determining eligibility for health services is for individuals to decide if their symptoms require medical attention, followed by navigation to care, which requires the knowledge and ability to enter appropriate services.^30^ Given the strong relationship between health-seeking behaviour and confidence in self-diagnosis,^40^ individuals’ identification of candidacy for long COVID may be impaired due to the lack of a clear illness trajectory and definition of the condition.^57^ When participants identified that their complaints required medical attention, they first contacted their GPs or, in some instances, the company/work healthcare professionals (identification). Subsequently, the lack of a proven treatment method^30^ and well-defined care pathways^28 29 57^ halted the process of establishing candidacy for care (navigation). The ease of entry into the health system was further complicated by alternating GPs and limited time for consultations (a general practice of the Dutch healthcare system), which hinder familiarity and effective patient–provider relationship to address the varied and multiple symptoms (permeability). In addition, symptoms such as ‘brain fog’, a common symptom of long COVID and characterised by difficulties with executive function, memory, and communication,^57^ impinged on individuals’ ability to accurately portray their symptoms and illness experience during encounters with health professionals, thereby reducing their ability to assert candidacy (appearances) and have this validated (adjudications). This has crucial implications for long COVID care access and acceptance of follow-up services or referrals to appropriate care (offers and resistance). Especially for Turkish and Moroccan ethnic minority participants who could not communicate in Dutch, ‘brain fog’ and the novelty of their symptoms exacerbated difficulties to clearly describe their symptoms, request for or get referred to care pathways, requiring them to depend on family members and children to advocate for their care. The use of family members and children to facilitate access to care has implications for patient privacy, and their understanding and translating of medical concepts and information which could potentially lead to miscommunication and/or misinformation. Perhaps, this challenge is more evident because the majority of Turkish and Moroccan ethnic minority study participants were first-generation migrants who might have had low Dutch language proficiency and digital literacy. The challenge to effective communication, particularly regarding limited or no national language proficiency, and the dependency on children to serve as translators in facilitating patients’ access to healthcare have been reported in several studies.^58^,^65^ This is potentially worse when people are less familiar with health services or have lower health literacy rates, such as among some ethnic minority populations.^39^ This dynamic process by which participants determined their eligibility for healthcare services, through continuous negotiations with their healthcare providers, highlights how vulnerabilities in accessing healthcare arise are influenced at multiple stages and differ between populations. This emphasises the crucial need for accessible, informative and tailored support systems to enhance access to information and care pathways for persons with long COVID and inform the public about current iterative evidence on long COVID and care.

Within the conceptual framework, upon entry, access is affected by the process of asserting candidacy during interactions with health professionals (appearances) and professional’s judgement about candidacy, influencing subsequent treatment (adjudications).^39^ While there were few reported overt experiences of double stigma and/or differential treatment based on ethnic minority background in this study, a general awareness of their minority position caused some Turkish and Moroccan ethnic minority participants to voice suspicions of differential treatment when they were not met with appropriate care. Layered on is the experience of stereotyping based on ethnicity or assumed migrant identity by health professionals, which further facilitated participants’ perception of differential treatment that impacted participants’ assertion of candidacy during their interactions with health professionals. Validations of candidacy by healthcare professionals are partially influenced by socially constructed notions about which patients are most likely to respond well and thus ‘deserve’ treatment as ‘ideal candidates’.^38 41^ These tie into the concept of stigma, as stigmatised individuals are less likely to be perceived as the ‘ideal candidate’ due to public stigma surrounding their identity. Thus, healthcare professionals need to recognise and address biases and inequities, especially towards ethnic minority populations who may face implicit healthcare and other social biases in accessing and receiving care.^37 39 66^ Such implicit biases could legitimise or adversely facilitate public stigma, especially for long COVID participants from ethnic minority populations, with further impacts on patients’ candidacy.^66^,^68^ In addition, while the findings show general difficulties in accessing care due to the lack of well-defined care pathways for long COVID, some Turkish and Moroccan ethnic minority participants faced additional difficulties such as limited access to information and resourceful and supportive networks, which compound existing health inequities.^59^

Personal relations and acquaintance with healthcare professionals with knowledge of long COVID were found to be key sources of information, access and referral to appropriate care sources outside of formal care pathways for long COVID participants. While the influence of social networks on peoples’ access to health information and navigation to care is more commonly reported among ethnic minority populations,^59^ in this study, both ethnic minority and the majority population used their social networks to facilitate candidacy and navigate access to care for their long COVID symptoms. Studies highlight the role of social networks and connections on health and social identity, which potentially influence people’ beliefs and health-seeking behaviour^27 42^ and shape candidacy. While the complexity and limited evidence on long COVID resulted in both healthcare professionals and participants being poorly informed on long COVID, other resourceful and informal networks provided an avenue to access information and care pathways for long COVID symptoms. This placed an immense burden on participants, particularly in the face of their long COVID illness, and potentially led them to access inappropriate information and care strategies to manage their symptoms, with critical impacts on their current and future health conditions.

For digitally literate participants, like findings from other studies,^17 69 70^ social networks, particularly online technologies and media, allowed long COVID patients to share and discuss symptoms, search for diagnosis and attempt to resolve identity dilemmas, which built a ‘*collective candidacy’* that facilitated their individual candidacy. For less digitally literate participants such as older or first-generation migrants, there is a need to harness localised and existing initiatives such as through the ‘*buurthuizen’* (neighbourhood houses), which are accessible community centres fostering social interaction, engagement and connections for residents including vulnerable persons. The importance of social connections and networks was more evident as non-Dutch speaking and more socially isolated participants experienced greater difficulties in navigating care within the Dutch health system. Consequently, social networks facilitated access to information sources such as C-support, which provided support and advice for long COVID. C-support was helpful in equipping participants with long COVID-related information such as specific referral requests to make from their GPs, which enabled them to better advocate for appropriate care and support for their illness. This emphasises the role strong social or professional networks can play on participants’ ability to assert candidacy in encounters with healthcare professionals in navigating access to care.

Furthermore, operating conditions surrounding the organisation and delivery of GP practices in the Netherlands, limited GP consultation time, bureaucracy of the health system, alternating GPs and participants’ perceptions of being misunderstood and the inadequate capacity of health professionals on long COVID influenced permeability and local production of candidacy. Consequently, Turkish and Moroccan ethnic minority persons contemplated seeking healthcare in their home countries where evidence shows they are known to often assess healthcare for specialist care and diagnostic assessments due to challenges in the operational conditions of the Dutch healthcare system, which court dissatisfaction with Dutch primary care.^71^,^74^ In the Dutch healthcare system, GPs are the gatekeepers to healthcare,^54^ functioning as key entry points, which requires time, trust and building strong relationships with participants to ease entry into the healthcare system. Several studies have also highlighted the importance of time, trust and building relationships between patients and GP services within the context of complex interventions to improve healthcare access.^75 76^ Participants felt that most GPs were uninformed about their illness, with some healthcare professionals assuming a psychological origin/cause for participants’ symptoms and thus contributing to their insecurities and self-stigma, particularly for patients with pre-existing health problems. Even with a diagnosis, access to referral and appropriate care was limited due to the novel nature (at the time) of long COVID and the subsequent paucity of literature and evidence on effective care and treatment pathways for long COVID. This greatly affected operating conditions for health professionals in establishing treatment and care plans for participants. Several studies have established the lack of well-defined care pathways or proven treatment methods that can be prescribed to all patients,^28^,^30^ which emphasises the need for clear operating conditions such as clinical and policy directions for novel and emergent conditions like long COVID. These would profoundly impact health professionals’ capacities through the availability of care pathways (permeability), their professional judgement about an individual’s candidacy, which influences subsequent treatments (adjudications) and patient and healthcare professional interactions and developments over time (local production of candidacy). While there is a need for health policy direction to enhance current operating conditions for treatment and care for long COVID, health professionals need to be equipped with evidence-based long COVID-related information as it emerges to facilitate local production of candidacy towards improving access to appropriate care and support for persons with long COVID. GPs could also facilitate social support for long COVID patients through sensitisation programmes to mitigate potential related stigma within the healthcare system and public if they are well informed. A recent qualitative study in the UK reported that ethnic minorities with long COVID experienced negative healthcare encounters at primary healthcare, which complicated access to secondary or specialist care with further access challenges and dissatisfaction with specialist care for long COVID.^66^ Particularly for ethnic and minority study participants, these challenges are further complicated by Dutch insurance costs and comparisons of the Dutch health system with other less bureaucratic health systems in other countries or their home countries. These potentially impact on patient and health professional interactions and participants’ acceptance or rejection of the follow-up services suggested for them by their GPs that require them to incur additional costs for the referral service or wait a longer period to access referral health services despite having health insurance (offers and resistance). Added on, as similarly reported in other studies, are pre-existing barriers to accessing healthcare services such as language difficulties, low literacy and digital literacy barriers, which further compound access to care.^77^,^80^ Training healthcare professionals such as nurse practitioners as case support managers for long COVID and building relationships with patients to provide practical support to navigate and access appropriate care could potentially reduce these structural barriers. There is also a need to sensitise the public on the feasibility of requesting longer consultation times for long COVID symptoms at GP services.

Moreover, the findings highlight the need to leverage social connections and networks to prevent and address stigma surrounding long COVID towards improving healthcare access to information and care through community sensitisation to facilitate patients’ candidacy in accessing care. Like findings of other studies,^81 82^ centrally structured multidisciplinary medical care could provide support in navigation and coordination of care to improve clinical outcomes for long COVID. While organisations such as C-support help patients navigate to care, providing a central care point of contact for long COVID linked to the GP system could reduce inequities in access to long COVID information and care. In addition, although C-support provided information and directions to care, the findings show that most non-Dutch-speaking participants recruited outside of C-support were not familiar with C-support. This shows the critical role that communication, particularly in the host country’s language and digital literacy, plays on access to health information. Patient organisations (both formal and informal) could be leveraged to reach and support sub-population groups by offering language and culturally concordant long COVID information and support, which may incrementally improve access to information and care among ethnic minority groups. In addition to growing calls for establishing nationwide registers of people with long COVID,^47^ collecting data on the lived experiences of various sub-populations could inform health policy and tailored implementation to improve access to long COVID care, especially for ethnic minority populations.

## Conclusions

The study findings show self and public stigma resulting from the debilitating illness and symptoms that persons with long COVID face, highlighting the need for public sensitisation. They also show that people with long COVID experience difficulties in accessing reliable information and healthcare resulting from several multifaceted factors related to candidacy. Social and pre-existing challenges and inequities such as communication (including digital) barriers and stereotyping by health professionals further compound ethnic minorities with long COVID's candidacy and experience of stigma. Leveraging social and community networks could potentially reach the diverse subpopulations with relevant long COVID-related information and care pathways in an iterative manner that improves access to information and care for long COVID.

## Supplementary material

10.1136/bmjopen-2024-094487 online supplemental file 1

10.1136/bmjopen-2024-094487 online supplemental file 2

10.1136/bmjopen-2024-094487 online supplemental file 3

## Data Availability

Data are available upon reasonable request.
