# Supplementary material for: Experiences of stigma and access to care among long COVID patients: a qualitative study in a multi-ethnic population in the Netherlands
Source: BMJ Open. 2025 Jun 6;15(6):e094487. doi: 10.1136/bmjopen-2024-094487 (PMC12164602; doi:10.1136/bmjopen-2024-094487)
Supplement: online supplemental file 1 [file bmjopen-15-6-s001.docx]

# Supplementary File 1: Interview guide

1. Can you tell me how the story of your COVID / long COVID illness started?

- When were you first ill with corona?
- When and how did you realize your symptoms were persisting?
- Did you relate this to that first corona infection?

2. What did you do when you realized your symptoms were persisting?

- When and where did you seek support? (assertion of candidacy)
  - Informal (e.g., friends, family, internet)
  - Formal (e.g., general practitioner, physiotherapists, psychologist)
- For what symptoms did you seek support? Why these?
- Was/is it clear to you where you could seek support? And what kind of support and care options were available?

3. How has long COVID affected your life and the way you see yourself?

- Has there been a change in your daily functioning? How has this change affected your self-image? (identity threat)
- Has there been a change in your interpersonal relationships? How has this change affected your self-image? (identity threat)
- Have you felt different or of other value than before / than others because of long covid? Have you felt embarrassed because of long covid? (internalized stigma)
- What brought about these feelings? What situations, what interactions (with whom) brought about these feelings? (enacted stigma)

4. How has the way you see yourself with long COVID affected when and where you sought support? (self-efficacy, assertion of candidacy)

- Is there a kind of public image of long covid in your community? in society? Can you describe it for me?
- How did this affect where you sought support? (anticipated stigma)
- Did anything else affect when and where you sought support? E.g. aspects of social identity, previous experiences? (double stigma)

5. What was your experience with [ healthcare professional] when telling them your symptoms?

- Did you feel heard? If so, what contributed to this? If no, why not? (testimonial (in)justice) (enacted stigma)
- Did [healthcare professional] link your symptoms to (long) COVID? (adjudications)
- Did they explain to you what long COVID is? Did they explain to you what to expect? (Can you describe the conversation?) Was it easy or difficult to understand the information? Why?
- Did they refer you or follow-up? (adjudications)
- Did you feel like something was missing? What?

6. What happened next? Can you describe other/subsequent contacts? (permeability, offers/resistance)

- What was your experience of these contacts?
- DK context: How did you get to know of the long COVID clinics?
- NL context: How did you get to know about C-Support?

7. How have your experiences with **long COVID care** affected the way you see yourself? And how you see your illness?

- How does this compare to other / previous experiences with care? (double stigma)

8. If you were to write our lessons learnt, in what ways would you say access to care for people with long COVID can be improved? In what ways can care for people with long COVID be improved?

- how can C-support be improved for people of ethnic minority background?

9. We are getting to the end of the interview. Thank you for sharing your experience with me. I will ask you to go through some basic characteristics with me. But before we check these I want to ask if you have anything to add?

Is there anything important you feel I missed to ask you?

10. Demographics

|  |  |
| --- | --- |
| Sex | Male, female, other |
| Age | ≥18-29, 30-39, 40-49, 50-59, 60-69, ≥ 70 |
| Country of birth | Morocco, Türkiye, The Netherlands |
| Country of birth mother | Morocco, Türkiye, The Netherlands |
| Country of birth father | Morocco, Türkiye, The Netherlands |
| Religion | Religious, not religious, prefer not to say |
| Educational level (highest obtained)  Work/study load prior to long covid. | Primary school, high school, MBO, HBO, WO  Fulltime, part-time, no (paid) work |
| Household composition | Living alone, living with partner or other adults, living with children (number/age) |
| Living conditions (type of housing) | Student housing or flat share, studio, flat or apartment, bungalow, semi-detached housing, detached housing. |
| Comorbid conditions | Diabetes, heart condition, previous stroke/apoplexy, hypertension, COPD/asthma, cancer, depressive or anxiety disorder, other? |
