# Supplementary material for: Experiences of stigma and access to care among long COVID patients: a qualitative study in a multi-ethnic population in the Netherlands
Source: BMJ Open. 2025 Jun 6;15(6):e094487. doi: 10.1136/bmjopen-2024-094487 (PMC12164602; doi:10.1136/bmjopen-2024-094487)
Supplement: online supplemental file 2 [file bmjopen-15-6-s002.docx]

| **Supplementary File 2: Participant quotes** | | |
| --- | --- | --- |
| **Theme** | **Sub-theme** | **Participant quotes** |
| Stigma | The experience of public and self-stigma | *“No, not so much my self-image. No. I do have a lot of support in my faith. I see things, trying things as a test so to speak.”* ***Participant 17 – MO***  *"Yes, was it corona then? Because you are positive, you were really sick too.... I was ashamed to prove that, how can you be sick for so long… Then it got better…then I had that recognition."* ***Participant 5 -MA*** |
|  | Stigma within the context of healthcare | *“I was accused by the family doctor.... because suddenly, I was going through all these things.... I suddenly had asthma, and everybody said you're getting demented or something... I went there because I also had problems with my back and peeing ... because I had been going to the GP a lot lately, and they didn't have anything, then they said, wrote that down that, sir it’s not real.”* ***Participant 12 - TU***  *“The GP also waits for you to come back there…. I assume they are looking hard for something because a lot of people suffer from that. So I assume that at some point there will be a solution, but.... I ’don't always feel it's taken seriously, no.."* ***Participant 1– Du***  *“Yes, you feel kind of abandoned, really abandoned. Because nobody understands you. Sometimes people around you at a certain point start thinking, "Yeah, ’don’t be silly."* ***Participant 4 - MO*** |
|  | Ethnic differences in experiences of stigma | *" I even have a speech impediment... normally, I speak fluent Dutch.... now I don’t manage quite well, but I have moments when I am searching. And then I say for fun I talk Turkish Dutch, that's more street language with the boys."* ***Participant 4 – TU***  *” I had contact with C-Support a few times…really good conversations... with the occupational therapist, they also told me that I should apply for this. But that all has to go through the family doctor, who has to make the referral letters.”* ***Participant 4 – MA*** |
| Access to care | Identification of candidacy and navigation to care | *"Those fatigue symptoms that have actually remained until now…. then after six months, I went to see the doctor again, because I had already done that a few times, but everyone said wait and see. And after six months I was sent to physical therapy…just outside the guideline. Because if you did it within so many months then it was covered by health insurance and now it wasn't, because it was again just too late."* ***Participant 1 – Du***  *“It was more about what is happening, and I think the doctor should have seen me in any case, if you are so sick with that. And he said, just looked at all the symptoms, and said, in principle we assume that you will recover, you are young and healthy.... he said, “Yes, usually if something lasts longer than 6 weeks you can speak of something chronic.” He said, “Come back then.” So, I did. And then he said, “Well, okay, the symptoms you still have, you could say you suffer from long COVID."* ***Participant 3 - DU***  *"So, people who don't speak the language fill in the gaps themselves or start asking and then get the wrong information, so wrong views. ... because where do you have to go to get the information? They usually have contact with a family doctor, not that he or she can speak Arabic or Turkish or whatever. But he or she can give the basic information but no more than that…" like "gosh, this is the medicine or not the medicine or go exercise or go for a walk. But no more than that."* ***Participant 5 – MA***  *” Uhm, the advice I would give, particularly to general practitioners, deviate from that 5-minute consultation hour. Take somebody seriously. Someone who has a complaint, they don't just come in…there are people who just need to be taken seriously…you have to make a double appointment... I find that with many things in the Netherlands, if I come on time and I have complaints, and you just give me time to only talk about one complaint, when I'm just really sitting with other complaints."* ***Participant 4 - MA***  *"So I was like, I think I should also just go and investigate myself what exactly is the reason why my back is bothering me so much then…but after I went to see the pulmonologist, she looked, there was nothing wrong actually, I should function. And yet I still feel it.... We were also a little further along in time, so there were more people trying to recover in different ways...Well we started searching on the Internet. But also talking to people who were also already suffering. And fortunately, I know a lot of people who suffer from it…I do know a lot of people, and the people who are then suffering from that, still, I did ask them specifically.... most of the people I've spoken to are women, who all work."* ***Participant 11 - TU*** |
|  | Permeability | *” If that family doctor, or that there's somebody.... that that just that binding just, of all those diseases, there should just be someone or an organization, that should just say hey these are just.... we're looking at what's missing, what the problem is, and we just need to address that… But I just had to convince the GP of my things…I get that too, because that GP who gets only 10 minutes of time from the care facility and he writes a tablet very quickly, painkiller."* ***Participant 12 – TU***  *“In April, I went back to the doctor again because I kept coughing. And I had painful joints, in addition to the symptoms I already had. And then I asked for a lung picture and an examination, whether I might have rheumatic complaints, inflammatory complaints … I was like if that GP doesn't look it up then I'm not getting the total picture, or nobody is getting that picture.... I had asked for a double interview...I had the idea that I could tell my story there... then she also said I don't really know…I didn't really know how to proceed.”* ***Participant 22 – Du***  *“At that moment, my family doctor was on vacation and there was a substitute family doctor. And he said I think you are just overworked, and there you have the number of the psychologist, go talk to a psychologist. …I tell him that may be, but I want a referral from him that I can use to do more than just go to a psychologist. So for example, I want to be able to go to a physio who specializes in this area and who can guide me through this… yes difficult, difficult. I said “well fine, but then I'll wait until my own family doctor is back and then I'll talk to him” … So then I got a kind of general referral letter.”* ***Participant 7 – Du*** |
|  | Appearances and adjudications | *” I think the very first point of entry is the family doctor.... because I think my condition didn't have to be so severe.... And if I have long COVID it's not called so for nothing huh, that means long-term COVID… initially I had recovered, I had absolutely no idea that this could come back this way. And the first few weeks that I went to the doctor, in February I had no idea that I had long COVID. And I don't think they did either.”* ***Participant 22 - DU***  *“I have a lot of back pain. It stayed after corona. I went to the doctor. [Because he] speaks Dutch I showed [on] the doctor's back with my hand: I have pain all here. [Pointing to lower back and middle back]. The doctor did nothing for me. Didn't give a painkiller…Also because I don't speak Dutch. My daughter-in-law went to call the doctor and he said he prescribed vitamins and no painkillers... ... after months my back pain didn't go away.… I talked to my family first and then went to the family doctor. The family doctor did nothing…a few weeks ago he referred me for an X-ray… I did go but was never referred to the hospital. I couldn't even go to my appointment that day…I had gone alone. Then the GP called my daughter-in-law to translate… There was no diagnosis."* ***Participant 25 – TU***  *” That cardiologist.... I also had sleep apnea before that.... I had already done a test once with one of those devices… that cardiologist said that's not their area of expertise.... So nobody listens to it. Then you have to be with your family doctor."* ***Participant 12 -TU*** |
|  | Offers and resistance | *”At that time, I went back to work I couldn’t do anything at all again, because I just felt very miserable. And when you have those relapses, it seems like you’re sick again. So, then it looks like you’re dying again. And that’s really, bad. So, in the end the doctor then referred me to that COVID ward in the [ZKHS]. And then I had to go all the way back to square one. So, then I really couldn’t do anything anymore… it was really very little what I was allowed to do in a day. That was really difficult… I had already lost that a few times [the deductible/own risk], I also have two children who I have to pay tuition fees for.”* ***Participant 1 – Du***  *” I was disoriented, I didn't know what to do. Then I contacted my GP again, and they sent me a referral for physio. And then I started at physio, and I also tried to walk outside every day... Then I went a few more times and then I thought no this is not good, I'm going to stop. Then I contacted the company doctor again and then I told them I stopped the physio, it's still not going so well, and then the company doctor said to me, but that's not good at all that…you just have to recover quietly and actually walk alone. So, build up slowly [recovery]. So, I was hearing different things everywhere, from all sides.”* ***Participant 6 - TU***  *“I don't know how to do that because we don't get reimbursed for that [therapy]…I came to an occupational therapist... I think I'll do everything once to see what's going to help me, then I'll make choices. And at the occupational therapist I thought what am I doing here? Then I went to that man, I said listen, this is not working, it doesn't give me energy, I can't get any further I want you to think of something else…He said I don't know if we will get reimbursed. When I told him, let me try it once to see if it's something for me, if that's feasible. I'd rather be walking outside than those rotten devices that drive me crazy.”* ***Participant 8_Du***  *“The occupational therapy was offered in February, … she said next time is the last time. I said oh no, no you can't. She said yes, but it's okay. I said yes, it's going well and I am in a rest now and I've learned schedules, but I have to go back to work later, and I think it's important to talk to someone who understands that occupational therapy part. I say you are the one for me who can help me create a different balance in my life and get back into a working mode. And then she said we have to apply for an extension, so you have to go back to that GP. I did that by phone. And that was ready for me again, and then there was that extension again. And I was very happy with that… I'm articulate right, and I think I know what I need…because I had used up my first piece and had not asked for an extension within a certain period. That's not good for the patients, because you don't know it's there, and you really need it.”* ***Participant 22 - DU***  *“The neurologist said do an MRI. I did that.... he gave me medication that I totally disagreed with…. I felt totally misunderstood by that neurologist. And I also had the feeling that he was just trying something out… it was very intense medication; I didn't like the side effects. It made me completely drowsy, the headache got even weirder or something…at that moment I didn't feel heard or anything.”* ***Participant 17 – MA***  *”I didn't feel like it anymore, I was tired, and then I didn't go. I couldn't handle it. It seemed like it was becoming chronic. Then the GP sent me to a sports doctor... especially with my lung infection the other day… I went to the doctor, and he said I don't hear anything crazy. … I looked at him, I said is that normal? He looked critically, it was a substitute, said it doesn't really look... he came to me, very shocked and says, I misjudged… there is no one from an agency [GP] who knows what or says I will coach you, I will take you all the way through that... no one takes responsibility, neither the GP nor the occupational therapist, nor the physio.”* ***Participant 4 - MA*** |
