# Supplementary material for: Experiences of stigma and access to care among long COVID patients: a qualitative study in a multi-ethnic population in the Netherlands
Source: BMJ Open. 2025 Jun 6;15(6):e094487. doi: 10.1136/bmjopen-2024-094487 (PMC12164602; doi:10.1136/bmjopen-2024-094487)
Supplement: Supplementary file 4 [file bmjopen-2024-094487.reviewer_comments.pdf]

PEER REVIEW HISTORY

BMJ Open publishes all reviews undertaken for accepted manuscripts. Reviewers are asked to complete a checklist review form and are provided with free text boxes to elaborate on their assessment. These free text comments are reproduced below.

ARTICLE DETAILS

Title (Provisional)

Experiences of stigma and access to care among long COVID patients: A Qualitative Study in a multi-ethnic population in The Netherlands

Authors

Nyaaba, Gertrude Nsorma; Torensma, Marieke; Goldschmidt, Maria Ingeborg; Nørredam, Marie; Moseholm, Ellen; Appelman, Brent; Rostila, Mikael; Tieleman, Peter; Biere-Rafi, Sara; Prins, Maria; Beune, Erik; Agyemang, Charles

---

VERSION 1 - REVIEW

**Reviewer**	**1**
**Name**	**Baz, Sarah**
**Affiliation**	**University of York, Health Sciences**
**Date**	**13-Dec-2024**
**COI**	**None**

Feedback for authors:

I think the paper could be shaped around looking at the relationship between stigma and candidacy in relation to long covid and accessing care more, this should be a key contribution and focus, potentially filling a gap in the literature.

Key message section - line 8. "scant literature on stigma and access to care from people’s lived experiences of long COVID." There are somewhat increasing publications in this area (particularly UK based studies), maybe there is emerging literature but still lacking in this area. Perhaps we still don't know the relationship between stigma and candidacy in relation to long covid - this could be a key gap the study addresses.

Example of UK study on long covid and candidacy - Title: Negotiation of collective and individual candidacy for long Covid healthcare in the early phases of the Covid-19 pandemic: Validated, diverted and rejected candidacy.

There may be more.

Lone 17 - what specific pre-existing challenges do ethnic minorities face? Give examples e.g., racism and discrimination.

Page 4 - statistics on hospitalized people, line 53 - how about non-hospitalised people, is there no data available in this area.

Nice to see Goffman cited in relation to stigma. Can his work be incorporated more here?

Page 6 - line 53 - can say here the study explores relationship between stigma and candidacy. Line 55 - should be "experiences of long covid".

Line 58 - Please add more background information about Netherlands population and its make-up, providing more context.

Excellent recruitment methods, a real strength of the study.

Data analysis section - Also deductive analysis took place here in terms of theoretical framework, so both inductive and deductive codes/analysis.

PPI in research section - Please provide more introductory information to the PPI group - when was it set up, was the PPI group involved in all research stages etc. This will be better in way of introducing the section rather than only having a table. It's not clear whether this project had a PPI group, it doesn't seem so from the table content. PPI groups are groups set up to feed into the research project - not the patients or participants being interviewed or researcher team members as described in table. E.g. in the second section in the table does "team members" refer to a PPI group or the research team - clarify please.

Results - table 1 - This would be better situated in the methods section, after explanation oof how many interviews ended up being conducted.

Page 13 - following statement "experience of strong socio-cultural norms regarding family responsibility within their communities." The participant quote doesn't show this, I think the interviewee is saying that it is a universal thing ("but I don't want to say that it is very different from the Dutch community"), everything can't be reduced to socio-cultural norms, this re-stigmatises ethnic minority communities depicting them as different. This more a gendered experience regarding society's gendered norms, which one could argue are manifested in all cultures.

Page 13 - loss of identity point - you could cite work on Long Covid and identity here, there are publications on long covid and biographical disruption.

Page 15 - how does discussion on health insurance and financial costs link back to candidacy - please explain.

Page 15-16 - point about people being dependent on children to book appointments and translate, could you refer to existing work and literature on this.

Lot of the findings are regarding structural barriers, systems and bureaucracy - which would be a good point to make in the discussion.

Discussion, page 2, first paragraph - "For ethnic minority populations, these challenges were further complicated by limited Dutch language proficiency and digital and/or health literacy."

Please add discrimination here as a barrier - particularly in relation to ethnicity and assumed migrant identity - a very interesting finding and contribution of the paper which should be included here and made more explicit.

What are the key contributions of this paper? - how does your paper develop stigma and candidacy theory and the relationship between them - this could be stated as a significant theoretical contribution with more details given. Has this been done before - maybe have a look back at the literature, particularly Long Covid literature.

Strengths and limitations - points on representativeness - qualitative research does not aim for this and through your methods you have reached populations that are often not heard in researched, which is commendable. I think we need to move beyond stating lack of representativeness as a weakness, as qualitative research does not typically set out for large representation and generalisability, it is more about the richness in data and people we are able to reach and share experiences of.

Overall, this is a great paper with much potential. Addressing the above comments will help further strengthen the paper and show its key contributions in this field.

Reviewer **2**

Name **Abejirinde, Ibukun-Oluwa Omolade**

Affiliation **University of Toronto Dalla Lana School of Public Health**

Date **10-Mar-2025**

COI **I have known the first author since we pursued our doctoral degree in the same program following which we have remained friends, being in touch once in a while. I thought carefully about the real or perceived conflict this may pose before agreeing to review this manuscript and only accepted the assignment with the confidence that I could fulfil this professional task to improve the quality and rigour of scientific endeavour.**

General Comments

Drawing from the concepts of stigma and candidacy (as a proxy for understanding access to care), this qualitative study comprising 23 interviews to explore the lived experiences of minority individuals in comparison with the majority (Dutch) in The Netherlands with long COVID.

The paper is generally well written and easy to follow, with sufficient level of detail outlining the rationale, approach, and methods (for data collection and analysis). There were a few things that needed clarification, and I felt that the intention to make comparisons from a cultural/ethnic perspective, may have resulted in generalised statements being made even when the evidence was not as clear cut. For example, a statement such as “For ethnic

minority populations, these challenges were further complicated by limited Dutch language proficiency and digital and/or health literacy" misses the nuance of intersections and risks conveying an impression that all ethnic minority groups have limited Dutch language proficiency and/or digital literacy- a stereotype that was refuted in some of the participants quotes.

See further points below:

Data Collection:

i) In reporting data collection, it is initially mentioned that three interviews were conducted online using video conferencing software, then later stated that the RAs conducted three online interviews each. With 2 RAs, this means 6 online interviews, not three.

ii) Could more insight be provided on how knowledge that data saturation had been reached was established and at which point of the process this was evident?

Data Synthesis and Analysis:

i) It is unclear the basis on which comparison between data from Dutch majority participants and ethnic minority participants was done- by comparing data strictly based on their ethnic identities? Keeping in mind that the authors affirm that the data gathered was considerably heterogenous and only 23 interviews were conducted (16 minority, 7 Dutch). As a reader, I am for example curious to know if the four participants (P3,8,15,18) who reported having had access timely referral were all Dutch or ethnic minorities.

Results:

i) I have concerns regarding the choice to label participants quotes when reporting the findings, based on their ethnic identities- Tu (Turkish), Mo (Moroccan) and Du (Dutch). This poses the risk of causing an overgeneralization of findings or a reinforcement of stereotypes, with participants individual experiences erased in favor of their group identity. Using group identity as the qualifier for the quotes further masks the recognition of any (other) intersecting social identities that may cumulatively explain everyone's experiences. Since authors report that "When explicitly asked about the experience of double stigma, however, most ethnic minority participants rejected the notion of being stigmatized because of their ethnicity", it further highlights the problematic nature of labelling experiences only based on individuals' ethnic identity, reinforcing social stereotypes that are harmful for visible minorities.

Discussion:

i) The authors may perhaps consider spending some space in the discussion section reflecting on if given the broader literature on health-related stigma and on access to care, the experiences noted in this study are unique to long-COVID or to the populations interviewed. This would present an opportunity to critically reflect on the broader implications re: health system responses for novel healthcare conditions where guidance

and knowledge even for professionals are scant and how local conditions (dimension 7 of the candidacy framework) including the policy landscape can be better prepared to respond to emergent conditions.

Other comments:

i. For the benefit of other researchers, perhaps the author can offer some insight into what their planned action was for the post-interview text message check-up if any of the interviewees had reported any health impacts following the

interviews.

ii. Minor grammatical errors were noted (e.g. a missing word in a sentence) in a couple of places.

VERSION 1 - AUTHOR RESPONSE

<table>
<tr><th colspan="3">A.Reviewer: 1 Dr. Sarah Baz, University of York</th></tr>
<tr><td>I think the paper could be shaped around looking at the relationship between stigma and candidacy in relation to long covid and accessing care more, this should be a key contribution and focus, potentially filling a gap in the literature.</td><td>This is not necessarily the focus of the paper as the Maclean A. et al (2023) has this direct focus. The focus in this paper is slightly different as it seeks to establish:

a) Is there is a relationship between stigma and long COVID? If yes, how? How is this applicable in the context of access to care?
b) Is there a relationship between candidacy and long COVID experience? If yes, how?
c) Do these differ between the majority population and the minority ethnic migrant populations in Netherlands?</td><td>All</td></tr>
</table>

	In essence, these are the key research questions that guide the structure and presentation of this paper.	
Key message section - line 8. "Scant literature on stigma and access to care from people's lived experiences of long COVID." There are somewhat increasing publications in this area (particularly UK based studies), maybe there is emerging literature but still lacking in this area. Perhaps we still don't know the relationship between stigma and candidacy in relation to long covid - this could be a key gap the study addresses.<br><br>Example of UK study on long covid and candidacy - Title: Negotiation of collective and individual candidacy for long Covid healthcare in the early phases of the Covid19 pandemic: Validated, diverted and rejected candidacy.<br><br>There may be more.	This section has been removed in accordance with the journal requirements.<br><br>Thanking for directing us to this UK study. Taking note of the findings of that paper, this paper is structured to address if there is a relationship between stigma and long covid and candidacy and long COVID and if this differs between populations.<br><br>They have slightly different focus but collectively provide a complementary understanding of the framework and its application to long COVID.<br><br>In lieu of your comment, the discussion has been revised to reflect the above.	2<br><br>21-25

Line 17 - what specific pre-existing challenges do ethnic minorities face? Give examples e.g., racism and discrimination.	This section has been removed in accordance with the journal requirements.	NA
Page 4 - statistics on hospitalized people, line 53 - how about non-hospitalised people, is there no data available in this area.	The statement provides statistics for both hospitalised (33-<br>8%) and non-hospitalised populations (13.7%) in the UK	4
Nice to see Goffman cited in relation to stigma. Can his work be incorporated more here?	Originally, this was done but due to word count limitation, Goffman is introduced here but is cited more in the discussion section. Citing more of his work here	NA

	would only further exceed our already high word count.	
Page 6 - line 53 - can say here the study explores relationship between stigma and candidacy.	The study is not focused on exploring the relationship between stigma and candidacy. It is more focused on the experience of long COVID and whether they experienced any form of stigma and how candidacy facilitated their access to health care during the illness experience.	NA
Line 55 - should be "experiences of long covid".	This has been revised.	6

Line 58 - Please add more background information about Netherlands population and its make-up, providing more context.	This has been revised to provide more clarity. Due to word count limitation, we are unable to provide an extensive description of the context.	6
Excellent recruitment methods, a real strength of the study.	Thank you for your commendation.	NA
Data analysis section - Also deductive analysis took place here in terms of theoretical framework, so both inductive and deductive codes/analysis.	This has been revised.	10
PPI in research section - Please provide more introductory information to the PPI group - when was it set up, was the PPI group involved in all research stages etc. This will be better in way of introducing the section rather than only having a table. It's not clear whether this project had a PPI group, it doesn't seem so from the table content. PPI groups are groups set up to feed into the research project - not the patients or participants being interviewed or researcher team members as described in table. E.g. in the second section in the table does "team members" refer to a PPI group or the research team - clarify please.	This has been revised. This study does not explicitly have a PPI group involved in the design of this study.	10

Results - table 1 - This would be better situated in the methods section, after explanation oof how many interviews ended up being conducted.	The table is a result of the recruitment methods and some of the characteristics derived from the data analysis so	9-10
	ideally, it should be in the results section. However, we have moved this to the methods as suggested.	
Page 13 - following statement "experience of strong socio-cultural norms regarding family responsibility within their communities." The participant quote doesn't show this, I think the interviewee is saying that it is a universal thing ("but I don't want to say that it is very different from the Dutch community"), everything can't be reduced to socio-cultural norms, this re-stigmatises ethnic minority communities depicting them as different. This more a gendered experience regarding society's gendered norms, which one could argue are manifested in all cultures.	This has been revised. The role of cultural and gendered expectations is clarified and highlighted.	13
Page 13 - loss of identity point - you could cite work on Long Covid and identity here, there are publications on long covid and biographical disruption.	It was quite interesting exploring the literature on that intersection i.e. biographical disruption + loss of identity + stigma+ long COVID experience. We have incorporated a bit of this into the discussion but are careful of our word count. Thank you for the direction.	22
Page 15 - how does discussion on health insurance and financial costs link back to candidacy - please explain.	This impacts on the last two stages of candidacy. They impact on participants' acceptance or rejection of the follow up services that might have been suggested for them at the	25
	GP that might require them to pay for the referral service or wait a long period to access referral health services despite having health insurance. They also have impact on patient and health professional interactions as the additional costs beyond health insurance could hinder the types	

	of referral services that GPs provide to their patients.	
Page 15-16 - point about people being dependent on children to book appointments and translate, could you refer to existing work and literature on this.	This has been incorporated into the discussion section	23
Lot of the findings are regarding structural barriers, systems and bureaucracy - which would be a good point to make in the discussion.	Due to word count limitations, we have focussed on critical points that are not adequately alluded to in existing literature.	NA
Discussion, page 2, first paragraph - "For ethnic minority populations, these challenges were further complicated by limited Dutch language proficiency and digital and/or health literacy." Please add discrimination here as a barrier - particularly in relation to ethnicity and assumed migrant identity - a very interesting finding and contribution of the paper which should be included here and made more explicit.	Thank you for the suggestion. This appears to be a stereotyping rather than straightforward discrimination. This stereotyping has been incorporated to reflect the assumed migrant identify point and how those influences (the perception of participants of) differential health care access and provision in the discussion.	23
What are the key contributions of this paper? - how does your paper develop stigma and candidacy theory and the relationship between them - this could be stated as a significant theoretical contribution with more details given. Has this been done before - maybe have a look back at the literature, particularly Long Covid literature.	Thanks for your comment. We have revised the abstract and discussion to take this into consideration. Again, due to word count limitations, we are unable to delve in depth into a discussion on this.	2-3

Strengths and limitations - points on representativeness - qualitative research does not aim for this and through your methods you have reached populations that are often not heard in researched, which is commendable. I think we need to move beyond stating lack of representativeness as a weakness, as qualitative research does not typically set out for large representation and generalisability, it is more about the richness in data and people we are able to reach and share experiences of.	Thank for your commendation on our work. We completely agree with the essence of qualitative data and how much our work provides rich data about a section of ethnic minority migrants. We aimed to show that our study findings may not be applicable to all long COVID populations that from which our study participants were drawn from. As show in our study methods, some people were eligible but unable to participate due to increased burden of their long COVID symptoms (see page 8).	2-3

	We have however revised our point under strengths and limitations to clearly reflect this point.	
Overall, this is a great paper with much potential. Addressing the above comments will help further strengthen the paper and show its key contributions in this field.	Thank you for your insightful comments and contributions	
C. Reviewer: 2<br>Dr. Ibukun-Oluwa Omolade Abejirinde, University of Toronto Dalla Lana School of Public Health, Trillium Health Partners Institute for Better Health		

General Comments<br><br>Drawing from the concepts of stigma and candidacy (as a proxy for understanding access to care), this qualitative study comprising 23 interviews to explore the lived experiences of minority individuals in comparison with the majority (Dutch) in The Netherlands with long COVID.<br><br>The paper is generally well written and easy to follow, with sufficient level of detail outlining the rationale, approach, and methods (for data collection and analysis). There were a few things that needed clarification, and I felt that the intention to make comparisons from a cultural/ethnic perspective, may have resulted in generalised statements being made even when the evidence was not as clear cut.	Thank you for your helpful comments and suggestions.<br><br>Our findings are only limited to our study participants (as stated in the limitations) and as such do not reflect other ethnic minority groups. However, this has been revised and been made more explicit throughout the document particularly considering that most ethnic minority participants are first generations migrants (See p23)	2

For example, a statement such as "For ethnic minority populations, these challenges were further complicated by limited Dutch language proficiency and digital and/or health literacy" misses the nuance of intersections and risks conveying an impression that all ethnic minority groups have limited Dutch language proficiency and/or digital literacy- a stereotype that was refuted in some of the participants quotes.		

Data Collection:<br><br>i) In reporting data collection, it is initially mentioned that three interviews were conducted online using video conferencing software, then later stated that the RAs conducted three online interviews each. With 2 RAs, this means 6 online interviews, not three.<br><br>ii) Could more insight be provided on how knowledge that data saturation had been reached was established and at which point of the process this was evident?	This has been revised. Each RA conducted 3 interviews and only 3 out of the total 23 interviews were conducted online by the MT, the shared co-author.<br><br>This has been included.	9<br><br>8 &<br><br>7
Data Synthesis and Analysis:	Yes, the data was primarily compared by ethnicity. Please see second paragraph of data analysis section	17

i) It is unclear the basis on which comparison between data from Dutch majority participants and ethnic minority participants was done- by comparing data strictly based on their ethnic identities? Keeping in mind that the authors affirm that the data gathered was considerably heterogenous and only 23 interviews were conducted (16 minority, 7 Dutch). As a reader, I am for example curious to know if the four participants (P3,8,15,18) who reported having had access timely referral were all Dutch or ethnic minorities.	We have provided more clarification to the four participants.	
Results:<br><br>i) I have concerns regarding the choice to label participants quotes when reporting the findings, based on their ethnic identities- Tu (Turkish), Mo (Moroccan) and Du (Dutch). This poses the risk of causing an overgeneralization of findings or a reinforcement of stereotypes, with participants individual experiences erased in favour of their group identity. Using group identity as the qualifier for the quotes further masks the recognition of any (other) intersecting social identities that may cumulatively explain everyone's experiences. Since authors report that "When explicitly asked about the experience of double stigma,	This is one of the key complexities that underpin research on ethnicity and health. Given that the study is focussed to identifying and understanding ethnic minority groups (specifically Turkish and Moroccan), it is relevant to let the reader understand and identify the differences based on the labelling of the quotes. Additional	NA

however, most ethnic minority participants rejected the notion of being stigmatized because of their	descriptors would potentially impact on the anonymity of the participants.<br><br>Secondly, three ethnic populations (Dutch, Turkish and Moroccan) were purposively included in the study. In addition, while we used a purposive sampling approach to	

ethnicity", it further highlights the problematic nature of labelling experiences only based on individuals' ethnic identity, reinforcing social stereotypes that are harmful for visible minorities.	ensure variation in ethnic origin, gender, educational level, migration status, and Dutch language proficiency, we primarily used ethnicity in our data analysis (Please see second paragraph of data analysis).<br><br>Lastly, qualitative study findings are about the shared experiences, the people eligible and recruited and the quality of the data so these findings do not necessarily apply to the larger ethnicity or illness populations. This is why we took time to provide clear information about our study population and recruitment methods. While the findings are not aimed at enhancing stereotypical behaviours, they are meant to guide the reader and provide some insights into key differences which could be leveraged to enhance and provide appropriate health care.	

Discussion:<br><br>i) The authors may perhaps consider spending some space in the discussion section reflecting on if given the broader literature on health-related stigma and on access to care, the experiences noted in this study are unique to long-COVID or to the populations interviewed. This would present an opportunity to critically reflect on the broader implications re: health system responses for novel healthcare conditions where guidance and knowledge even for professionals are scant and how local conditions (dimension 7 of the candidacy framework) including the policy landscape can be better prepared to respond to emergent conditions.	Thank you for your insightful comment. We have revised the discussion to reflect this. We are however limited by our word count in going in depth on the point as we have touched on dimension 7 throughout the discussion.	25
Other comments:<br><br>i. For the benefit of other researchers, perhaps the author can offer some insight into what their planned action was for the post-interview text message check-up if any of the interviewees had reported any health impacts following the interviews.<br><br>ii. Minor grammatical errors were noted (e.g. a missing word in a sentence) in a couple of places.	This was to check-up on any health impacts experienced following their interviews and if any, to provide some guidance and signpost to appropriate health care for further support. This has been included in the revised manuscript. Furthermore, it is good research conduct to show empathy and care towards study participants as the interview was potentially very taxing for the participants with long COVID.<br><br>Minor errors identified and addressed throughout.	9

VERSION 2 - REVIEW

**Reviewer**	**2**
**Name**	**Abejirinde, Ibukun-Oluwa Omolade**

**Affiliation**	**University of Toronto Dalla Lana School of Public Health**
**Date**	**12-May-2025**
**COI**	

No further comments
